# Full-Length Transcriptome Sequencing and Comparative Transcriptomic Analyses Provide Comprehensive Insight into Molecular Mechanisms of Flavonoid Metabolites Biosynthesis in *Styphnolobium japonicum*

**DOI:** 10.3390/genes15030329

**Published:** 2024-03-03

**Authors:** Miao Wu, Yu Zhang, Peng Guo, Huiyuan Liu, Linkui Xia, Mengyuan Wang, Chuqi Zeng, Hongwei Wang, Fude Shang

**Affiliations:** 1 College of Life Sciences and Engineering, Henan University of Urban Construction, Pingdingshan 467044, China; wumiao@hncj.edu.cn (M.W.);; 2 Henan Engineering Research Center for Osmanthus Germplasm Innovation and Resource Utilization, Henan Agricultural University, Zhengzhou 450002, Chinawhwcas@163.com (H.W.)

**Keywords:** *Styphnolobium japonicum*, flavonoid biosynthesis, full-length transcriptome, gene expression, molecular mechanisms

## Abstract

*Styphnolobium japonicum* L. is a commonly consumed plant in China, known for its medicinal and nutritional benefits. This study focuses on the medicinal properties influenced by flavonoid metabolites, which vary during flower development. Utilizing full-length transcriptome sequencing on *S. japonicum* flowers, we observed changes in gene expression levels as the flowers progressed through growth stages. During stages S1 and S2, key genes related to flavonoid synthesis (*PAL*, *4CL*, *CHS*, *F3H*, etc.) exhibited heightened expression. A weighted gene co-expression network analysis (WGCNA) identified regulatory genes (*MYB*, *bHLH*, *WRKY*) potentially involved in the regulatory network with flavonoid biosynthesis-related genes. Our findings propose a regulatory mechanism for flavonoid synthesis in *S. japonicum* flowers, elucidating the genetic underpinnings of this process. The identified candidate genes present opportunities for genetic enhancements in *S. japonicum*, offering insights into potential applications for improving its medicinal attributes.

## 1. Introduction

*Styphnolobium japonicum* L., commonly referred to as the Chinese scholar tree, is extensively cultivated in East Asia and recognized for its multifaceted utility. As a leguminous tree native to China, it serves as a traditional industrial crop [1,2,3]. Renowned for its ornamental, medicinal, and edible characteristics, *S. japonicum* holds a significant place in various applications. The flowers of *S. japonicum*, considered a traditional medicinal plant, exhibit antioxidant and antibacterial properties, making them a valuable component in traditional Chinese medicine [4]. The *S. japonicum* flower, also known as the Huai flower, holds dual significance as both a medicinal and edible plant material. Widely consumed in China, it is utilized as a preservative, a color and flavor additive, and an edible component in food production [5,6,7].

Previous studies have demonstrated that *S. japonicum* flower buds exhibit the potential to alleviate oxidative stress and address various clinical diseases [8,9,10]. Owing to active ingredients that primarily comprise secondary metabolites, including flavonoids, volatile oils, and polysaccharides [11,12,13]. Recent research has highlighted a substantial content of flavonoids in *S. japonicum* flowers, and these flavonoids, with a history of use in traditional Chinese herbal medicine, are known for their potential biological activities [14,15,16]. In addition to flavonoid components, *S. japonicum* flowers contain various other constituents, such as amino acids, dietary fiber, glycosides, polysaccharides, terpenoids, and alkaloids. Due to their high nutritional and health value, these flowers find widespread application in edible food products [4,17].

Earlier studies indicated that the harvest time significantly influences the essential oil components of *S. japonicum*, with the highest content observed during the flower bud stage [18]. Flavonoid content varies across different maturation stages of *S. japonicum* flowers, impacting antioxidant and tyrosinase inhibitory activities [2]. Flavonoids and phenolic acids emerge as major metabolites in *S. japonicum* flowers, and their content undergoes alterations throughout the flower development process [19]. While existing studies have primarily focused on the activities and composition of components at different development stages of *S. japonicum* flowers, there is a noticeable gap in the literature regarding gene expression and regulatory networks associated with the biosynthesis of these metabolites in different flower stages. This stands in contrast to the extensive elucidation of biosynthesis pathways and key enzymes in other plants.

Recently, the rapid development of Next-Generation Sequencing technology (NGS), transcriptomics, and high-throughput sequencing provided high throughput and accuracy effective for widely identifying and characterizing genes in many non-model plants. However, based on the NGS, the RNA-seq only generated short reads with amplification bias which limits the acquisition of high-quality full-length transcript sequences and probably resulted in the loss of some vital information. The single-molecule real-time sequencing (SMRT) technology of the PacBio RS system provided a new platform with third-generation sequencing, which generates long read lengths, high quality, and a low degree of bias. Therefore, SMRT sequencing technology is an effective and reliable strategy for generating more complete and comprehensive genetic information.

In this study, the full-length transcriptome of the *S. japonicum* flower was sequenced using SMRT sequencing, and was further used as a reference for the accurate analysis of the transcriptional regulation of biosynthesis of secondary metabolites in the flower of *S. japonicum*. The global gene expressions in different-stage flowers were performed, and those involved in the secondary metabolism biosynthesis were specifically analyzed. In addition, the related genes of the regulatory networks of biosynthesis pathways in the flower of *S. japonicum* were identified through weighted gene co-expression network analysis (WGCNA). This study will provide a basis for the genetic information of secondary metabolite synthesis in *S. japonicum* during flower bud development.

The rapid advancement of Next-Generation Sequencing (NGS) technology, transcriptomics, and high-throughput sequencing has facilitated high throughput and accurate identification and characterization of genes in numerous non-model plants. In this study, we utilized SMRT sequencing to sequence the full-length transcriptome of the *S. japonicum* flower. This dataset was subsequently employed as a reference for the accurate analysis of the transcriptional regulation of flavonoid metabolite biosynthesis in *S. japonicum* flowers. Additionally, related genes in the regulatory networks of biosynthesis pathways in *S. japonicum* flowers were identified using the weighted gene co-expression network analysis (WGCNA). This study lays the groundwork for understanding the genetic information underlying flavonoid metabolite synthesis in *S. japonicum* during flower bud development.

## 2. Materials and Methods

### 2.1. Plant Materials Preparation and Collection

The flowers of *S. japonicum* were obtained from the Henan University of Urban Construction (33°46′ N, 113°11′ E), Pingdingshan, China. Based on the growth appearance, we categorized flower development into four stages: the young-bud stage (S1), early-bud stage (S2), late-bud stage (S3), and full-bloomed stage (S4). All collected samples in vitro were rapidly frozen using liquid nitrogen and subsequently stored at −80 °C. Each stage necessitated three biological replicates, with a minimum of 1 g of samples collected from three individual trees within each stage.

### 2.2. Total RNA Extraction

The total RNA from *S. japonicum* flowers was isolated using the RNAprep Pure Plant Plus kit (TIANGEN Biotech, Bejinig, China) following the manufacturer’s instructions. The concentration of RNA was determined using a Nanodrop micro-spectrophotometer (Thermo Fisher Scientific Inc., Waltham, MA, USA). The purity and integrity of the RNA were assessed using an Agilent 2100 bioanalyzer (Agilent, Santa Clara, CA, USA). RNA samples that met the criteria for library construction and sequencing were retained for a subsequent analysis.

### 2.3. RNA-seq Library Preparation and Sequencing

The qualified RNA samples extracted from flowers at each of the four stages underwent enrichment with magnetic beads containing Oligo (dT), and three biological replicates were prepared for each sample. To break the mRNA into short fragments, a fragmentation buffer was utilized. The first-strand cDNA was synthesized using a random hexamer primer and M-MuLV reverse transcriptase. Subsequently, the second strand of cDNA was synthesized with DNA polymerase I and RNase H. The resulting cDNA fragments were quantitatively amplified via PCR and purified using AMPure XP beads to obtain a library. To ensure the quality of the constructed library, the Qubit 2.0, Agilent 2100 bioanalyzer, and qRT-PCR were employed. The libraries were collected and sequenced on the Illumina NovaSeq 6000 platform. Following sequencing, clean reads were obtained by further filtering out raw reads with adaptors, low-quality sequences, and unknown nucleotides exceeding 10%. Quality control analyses were conducted to assess the Q30 and GC contents of the clean reads.

### 2.4. SMRT Sequencing Library Construction and Sequencing

For full-length transcriptome sequencing, libraries were constructed using total RNA obtained by combining equal amounts from flowers at all four stages (S1, S2, S3, and S4). Enriched mRNA was extracted and utilized for the synthesis of full-length cDNA employing the Clontech SMARTer PCR cDNA Synthesis Kit (Clontech, Mountain View, CA, USA). The resulting cDNA was then subjected to PCR amplification for library construction. Subsequently, the libraries were sequenced on a PacBio Sequel II system, and the Iso-Seq data were generated and made available through the National Genomics Data Center (https://ngdc.cncb.ac.cn/, accessed on 13 November 2025) with the BioProject accession number PRJCA021261.

### 2.5. Processing and Error Correction of PacBio Iso-Seq Reads

The sequence data underwent processing using SMRTlink 5.0 software following the standard Iso-Seq protocol. High-quality circular consensus sequences (CCS) were obtained from subread BAM files with specific parameters: min_length 200, max_drop_fraction 0.8, no_polish TRUE, min_zscore -9999, min_passes 1, min_predicted_accuracy 0.8, max_length 18000. The resulting CCS.BAM files were then categorized into full-length and non-full-length reads by disregarding poly(A) false reads and applying a minimum sequence length of 200. The full-length non-chimeric (FLNC) transcripts were isolated based on the presence of 5’ adapter sequences, 3’ adapter sequences, and poly(A) tails. Subsequently, the FLNC reads underwent clustering to generate consensus sequences, which were further refined using the LoRDEC (v0.7) software [20]. To eliminate redundancy, CD-HIT (v4.6.8) software was employed, resulting in the acquisition of the final isoform sequences for the subsequent analysis [21].

### 2.6. Transcripts’ Functional Annotations

Gene functions were annotated using BLAST analysis, employing an e-value threshold of “1 × 10^−5^”, against several databases, including NCBI non-redundant protein sequences (Nr), NCBI nucleotide sequences (Nt), Clusters of Orthologous Groups of proteins/euKaryotic Ortholog Groups of proteins (COG/KOG), SwissProt, Kyoto Encyclopedia of Genes and Genomes Ortholog database (KEGG), and gene ontology (GO). Family identification was conducted using the Hmmscan (3.1) software against the protein family (Pfam) database. The prediction of transcription factors (TFs) was carried out using the iTAK (1.7a) software [22].

### 2.7. Gene Expression Quantification

To quantify gene expressions, the RNA-seq clean reads were aligned to the full-length transcripts generated using SMRT sequencing. Bowtie2 was employed for aligning the reads obtained from each sample with the transcript sequences. Subsequently, RSEM (v1.3.0) software was utilized to statistically analyze the comparison results of clean reads’ alignment from Bowtie2 [23]. Considering the impact of sequencing depth and gene length on the obtained read counts, the fragments per kilobase of transcript per million mapped reads (FPKM) was utilized for calculating and quantifying each gene’s expression level. This metric could be directly applied to discern differences in gene expression between samples from different groups. Following gene expression quantification, differentially expressed genes (DEGs) between the comparison groups were identified using DEGs’ analysis using DESeq2 (v1.12.0) software [24]. Genes meeting the criteria |log2FC| ≥ 1 and adjusted *p*-value < 0.05 were deemed significant DEGs and included in the analysis.

### 2.8. GO and KEGG Enrichment Analysis

To elucidate the manifestation of differences in gene function among samples, the distribution of differentially expressed genes (DEGs) in gene ontology (GO) was analyzed using enrichment analysis [25]. GO enrichment analysis employed hypergeometric distribution, and corrected *p*-values were obtained through hypothesis verification to determine whether DEGs were enriched in specific GO terms. In this investigation, GOseq (v1.10.0) software was utilized for GO enrichment analysis. It calculates the probability of DEGs enriching in their corresponding GO terms based on Wallenius non-central hypergeometric distribution. Additionally, KEGG enrichment analysis was performed to identify the most critical biochemical metabolic and signal transduction pathways associated with DEGs. The KOBAS (v3.0) software was employed for pathway enrichment analysis, utilizing the hypergeometric test to identify significantly enriched pathways among DEGs [26].

### 2.9. Gene Co-Expression Analysis

The weighted gene co-expression network analysis (WGCNA) was performed using the R package WGCNA to construct a co-expression network with the differentially expressed genes (DEGs) and identify hub genes. Utilizing gene expression patterns across the four flower development stages, expressed genes were categorized into distinct modules, and the associations between these modules and related traits were calculated. The Cytoscape (3.5.1) software was employed to visualize the gene co-expression network and identify the hub genes.

## 3. Results

### 3.1. Analysis of S. japonicum Flower Full-Length Transcripts

**Figure 1:**
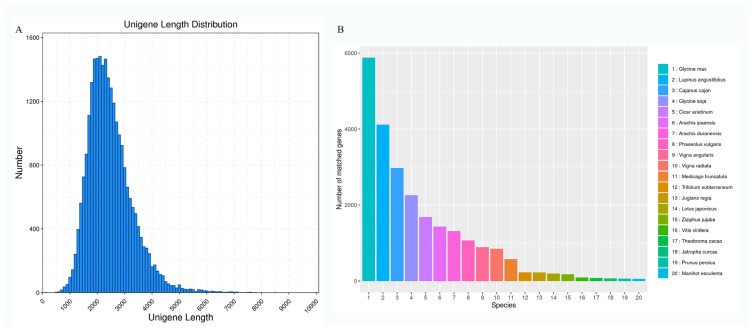
The distribution of transcripts’ lengths and species with annotated unigenes. (**A**) The distribution of transcripts’ lengths in the flower of *S. japonicum*. (**B**) The distribution of annotated species with all acquired unigenes.

To elucidate the molecular basis of *S. japonicum* flower development, high-quality RNA extracted from flower samples at four stages (S1, S2, S3, and S4) was sequenced using the PacBio Sequel platform. A total of 103.9 GB of raw data was generated. After eliminating connectors and sequences with lengths less than 50 bp detected in the raw data, 21,260,942 subread sequences were obtained with an average length of 2516 bp and an N50 of 2608 bp (Table S1). This included 629,630 CCS sequences with an average length of 2663 bp and an N50 of 2706 (Table S2). Post-clustering of CCS reads resulted in 525,408 full-length non-chimeric (FLNC) reads, accounting for 83.45% (Table S3). Subsequently, low-quality isoforms of FLNC sequences and redundant transcripts were removed, resulting in 53,281 transcripts with length distributions of <1 kb, 1–2 kb, 2–3 kb, and >3 kb (274, 17,057, 25,957, and 9993, respectively) (Figure 1A). Further sequence clustering and removal of redundant and similar sequences using CD-HIT yielded a total of 25,564 high-quality full-length unigenes.

### 3.2. Gene Function Annotation

The high-quality unigenes of *S. japonicum* were perfectly annotated with the Nr, Nt, Pfam, COG, GO, Swiss-prot, and KEGG databases (Table 1). A total of 25,427 genes were annotated in at least one database, with 13,150 genes being annotated in all databases. The results of sequence alignment with the NR database indicated the successful annotation of 25,261 genes. Similarity analysis revealed three major species with the highest homology: Glycine max, Lupinus angustifolius, and Cajanus cajan (Figure 1B). Moreover, 16,474 genes were annotated in the COG database, categorized into 25 major functional clusters (Figure S1). The three largest categories were ‘general function prediction only’, ‘signal transduction mechanisms’, and ‘posttranslational modification, protein turnover, chaperones’. Additionally, 18,952 genes were assigned to 51 GO terms, distributed across three major categories: biological process, molecular function, and cellular component (Figure S2). In the KEGG database, 25,175 genes were assigned, with 7250 genes classified into 33 pathways (Figure S3).

### 3.3. Gene Expression Analysis of S. japonicum Flower

Utilizing the RSEM (1.3.0) software to calculate fragments per kilobase million (FPKM) values for the detected genes, we identified 24,248, 22,847, 22,125, and 21,721 genes in the S1, S2, S3, and S4 flower stages, respectively. The gene expression analysis for each group revealed a total of 18,534 genes detected across all four stages. Additionally, 209 genes were specifically expressed in the S1 group, 86 in the S2 group, 163 in the S3 group, and 186 in the S4 group (Figure S4).

The differentially expressed analysis among four stages of flowers of *S. japonicum* showed that six comparison groups were constructed, which included S1-vs.-S2, S1-vs.-S3, S1-vs.-S4, S2-vs.-S3, S2-vs.-S4, S3-vs.-S4. Based on the statistical result, we showed that 7964 DEGs were collected in the comparison group of S1-vs.-S2, with 5048 genes upregulated and 2916 downregulated (Table 2), while in the S1-vs.-S3 comparison group, 11,824 DEGs (6570 upregulated and 5254 downregulated) (Table 2) were detected. In the S1-vs.-S4 comparison group, 12,768 DEGs (7576 upregulated and 5192 downregulated) (Table 2). The other comparison groups such as S2-vs.-S3, S2-vs.-S4, and S3-vs.-S4 were detected at 12,804 (6558 upregulated and 6246 downregulated), 12,278 (6895 upregulated and 5383 downregulated) and 13,215 DEGs (7439 upregulated and 5776 downregulated), respectively (Table 2).

### 3.4. Functional Enrichment Analysis of DEGs

**Figure 2:**
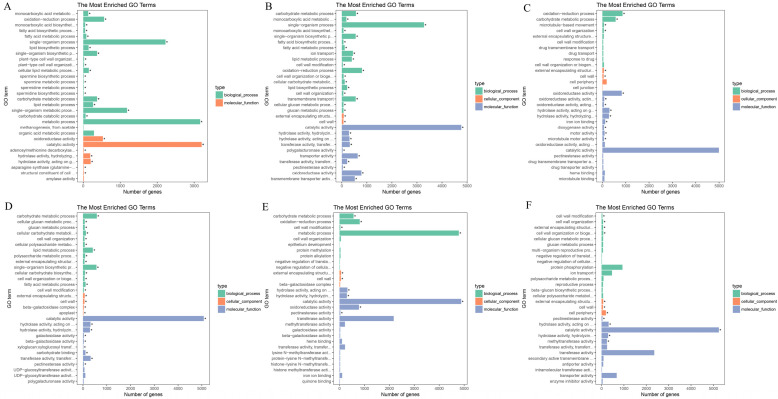
GO functional enrichment analysis of DEGs. The bars marked with “*” indicate significantly enriched terms. (**A**) The GO functional enrichment analysis of S1-vs.-S2 comparison group. (**B**) The GO functional enrichment analysis of S1-vs.-S3 comparison group. (**C**) The GO functional enrichment analysis of S1-vs.-S4 comparison group. (**D**) The GO functional enrichment analysis of S2-vs.-S3 comparison group. (**E**) The GO functional enrichment analysis of S2-vs.-S4 comparison group. (**F**) The GO functional enrichment analysis of S3-vs.-S4 comparison group. The *Y*-axis indicates the enriched GO term; the *X*-axis indicates the number of DEGs enriched in each GO term.

To explore the functions of differentially expressed genes (DEGs) across various flower development stages in *S. japonicum*, gene ontology (GO) analysis was conducted. In the S1-vs.-S2 comparison group, DEGs were notably enriched in 28 GO terms, predominantly falling within two categories: biological process and molecular function. Within the biological process category, numerous DEGs were associated with subcategories such as ‘metabolic process’, ‘single-organism process’, and ‘single-organism biosynthetic process’ (Figure 2A). In the molecular function category, DEGs were enriched in subcategories like ‘catalytic activity’, ‘oxidoreductase activity’, and ‘hydrolase activity, acting on glycosyl bonds’ (Figure 2A).

In the S1-vs.-S3 comparison group, DEGs were significantly enriched in 72 GO terms spanning three categories: biological process, cellular process, and molecular function. The analysis indicated that the majority of DEGs were enriched in the biological process and molecular function categories (Figure 2B). Notably, within the biological process category, DEGs were found in subcategories like ‘single-organism process’, ‘oxidation−reduction process’, and ‘carbohydrate metabolic process’ (Figure 2B).

For the S1-vs.-S4 comparison group, a considerable portion of DEGs was enriched in subcategories such as ‘catalytic activity’, ‘oxidation−reduction process’, and ‘oxidoreductase activity’ (Figure 2C). In the comparison groups of S2-vs.-S3 and S3-vs.-S4, the majority of DEGs were found in the subcategory of ‘catalytic activity’ (Figure 2D,F). In the S2-vs.-S4 comparison group, besides ‘catalytic activity’, DEGs were also enriched in the ‘metabolic process’ subcategory (Figure 2E).

**Figure 3:**
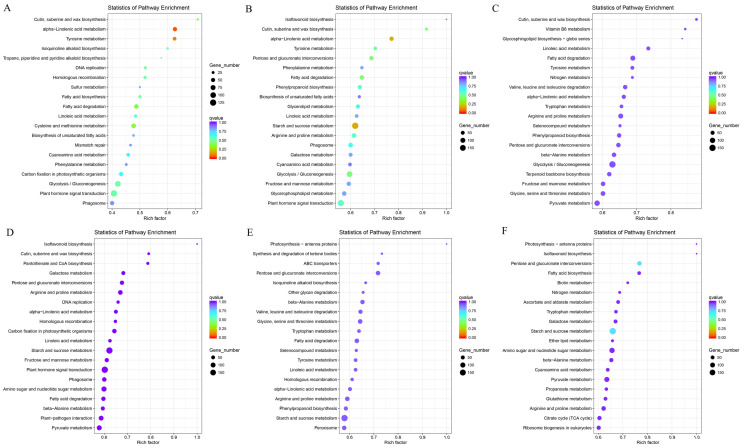
KEGG pathway enrichment analysis of DEGs. (**A**) The S1-vs.-S2 comparison group. (**B**) The S1-vs.-S3 comparison group. (**C**) The S1-vs.-S4 comparison group. (**D**) The S2-vs.-S3 comparison group. (**E**) The S2-vs.-S4 comparison group. (**F**) The S3-vs.-S4 comparison group. The *Y*-axis indicates the KEGG pathway; the *X*-axis indicates the rich factor. The bubble size indicates the number of DEGs of the pathway, and the color bar indicates the q-value.

In the Kyoto Encyclopedia of Genes’ and Genomes’ (KEGGs’) pathway analysis, 114 and 116 pathways were identified in the S1-vs.-S2 and S2-vs.-S4 comparison groups, respectively. Moreover, 117 pathways were identified in the S1-vs.-S3, S1-vs.-S4, S2-vs.-S3, and S3-vs.-S4 comparison groups. The top 20 enriched pathways in each comparison group revealed that many DEGs were enriched in pathways related to ‘Plant hormone signal transduction’, ‘Starch and sucrose metabolism’, ‘Phenylpropanoid biosynthesis’, ‘Isoflavonoid biosynthesis’, ‘Arginine and proline metabolism’, and various fatty acid metabolism-related pathways (Figure 3A,B,D,E). These findings suggest a potential correlation between DEG functions and flower development stages.

### 3.5. Cluster Analysis of DEGs

To systematically evaluate the concordance between gene expression and flower development in *S. japonicum*, the expression of differentially expressed genes (DEGs) across the flower time series was analyzed using fuzzy c-means clustering. A total of 20 distinct clusters were obtained, each displaying different gene expression patterns (refer to Table S4). Among these clusters, Cluster 2, 5, 17, and 18 indicated that the genes collected reached their highest expression levels during the S1 stage, gradually decreasing over time in Clusters 2 and 18. Clusters 7, 9, 10, 11, 13, and 15 represented genes that attained their highest expression levels in the S2 stage. Conversely, Clusters 1, 6, 8, 12, and 16 indicated the highest expression of genes during the S3 stage. The remaining clusters (3, 4, 14, 19, and 20) comprised genes that exhibited their highest expression in the S4 stage.

KEGG pathway enrichment analysis of clusters with higher expression levels during S1 and S2 stages revealed significant enrichments. In Cluster 2, pathways such as ‘Butanoate metabolism’, ‘Valine, leucine and isoleucine biosynthesis’, ‘Linoleic acid metabolism’, ‘Flavonoid biosynthesis’, and ‘Synthesis and degradation of ketone bodies’ were significantly enriched (refer to Table 3). In Cluster 5, pathways including ‘α-Linolenic acid metabolism’, ‘Isoflavonoid biosynthesis’, ‘Ubiquinone and other terpenoid-quinone biosynthesis’, ‘Glycolysis/Gluconeogenesis’, ‘Plant hormone signal transduction’, ‘Fatty acid degradation’, ‘Cutin, suberine and wax biosynthesis’, ‘Peroxisome’, and ‘Fatty acid degradation’ showed significant enrichment (refer to Table 3). Cluster 7 revealed enrichment in pathways such as ‘Porphyrin and chlorophyll metabolism’, ‘Diterpenoid biosynthesis’, ‘Vitamin B6 metabolism’, ‘Ubiquinone and other terpenoid-quinone biosynthesis’, and ‘Phenylalanine, tyrosine and tryptophan biosynthesis’ (refer to Table 3). In Cluster 9, pathways including ‘Linoleic acid metabolism’, ‘Isoquinoline alkaloid biosynthesis’, ‘Flavonoid biosynthesis’, ‘Other glycan degradation’, ‘Sphingolipid metabolism’, and ‘Carbon fixation in photosynthetic organisms’ were significantly enriched (refer to Table 3).

Furthermore, significant enrichment was observed in Clusters 13 and 18. Cluster 13 showed enrichment in pathways such as ‘Fatty acid biosynthesis’, ‘Biotin metabolism’, ‘Starch and sucrose metabolism’, ‘Amino sugar and nucleotide sugar metabolism’, ‘Steroid biosynthesis’, and ‘Tryptophan metabolism’ (refer to Table 3). In Cluster 18, pathways including ‘Linoleic acid metabolism’, ‘DNA replication’, ‘α-Linolenic acid metabolism’, ‘Spliceosome’, ‘Flavonoid biosynthesis’, ‘Phenylpropanoid biosynthesis’, ‘Pyrimidine metabolism’, and ‘Sesquiterpenoid and triterpenoid biosynthesis’ exhibited significant enrichment (refer to Table 3). These findings indicate that genes with higher expression levels in clustered patterns play vital roles in different flower development stages.

### 3.6. Expression of Flavonoid Synthesis-Related Genes in the Flower of S. japonicum

**Figure 4:**
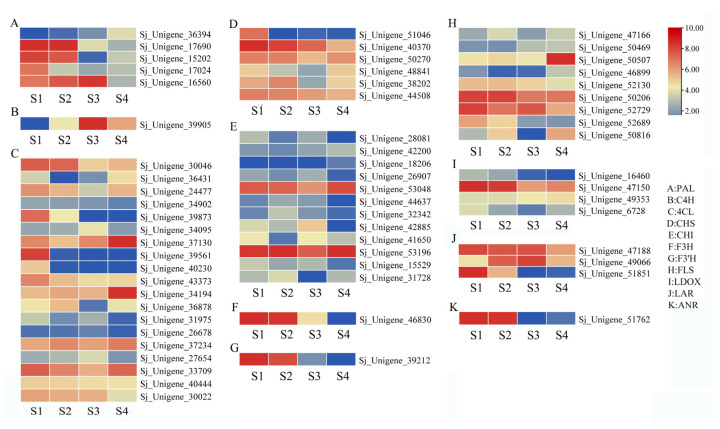
The expression analysis of flavonoid synthesis-related genes. (**A**) *PAL* gene. (**B**) *C4H* gene. (**C**) *4CL* gene. (**D**) *CHS* gene. (**E**) *CHI* gene. (**F**) *F3H* gene. (**G**) *F3′H* gene. (**H**) *FLS* gene. (**I**) *LDOX* gene. (**J**) *LAR* gene. (**K**) *ANR* gene. The color bar indicates the normalized log2 transformed fold change value.

In this study, the majority of genes detected were related to flavonoid synthesis, and their expression levels varied across different flower development stages. Flavonoids play diverse functional roles in both plants and humans, and their synthesis involves multiple steps within related pathways. For instance, the initial three steps in the phenylpropanoid pathway encompass *PAL*, *C4H*, and *4CL* genes. The expression patterns of these genes revealed that the *C4H* gene was upregulated with the progression of flower development stages (Figure 4B). Conversely, most *PAL* and *4CL* genes exhibited upregulation in the S1 stage, gradually decreasing as the flower development period advanced (Figure 4A,C).

Furthermore, other genes involved in the various branches of flavonoid synthesis were identified and exhibited differential expression across the four flower stages. Results indicated that many of these collected genes were upregulated in the S1 and S2 stages compared to the other two stages. Examples include *CHS*, *F3H*, *F3′H*, *LDOX*, *LAR*, and *ANR* genes (Figure 4D,F,G,I–K). On the other hand, the expression patterns of *CHI* and *FLS* genes showed variations among the four stages (Figure 4E,H). The identified differentially expressed genes (DEGs) provide valuable insights into the mechanistic aspects of specific flower development stages over time.

### 3.7. Construction of Gene Co-Expression Network

**Figure 5:**
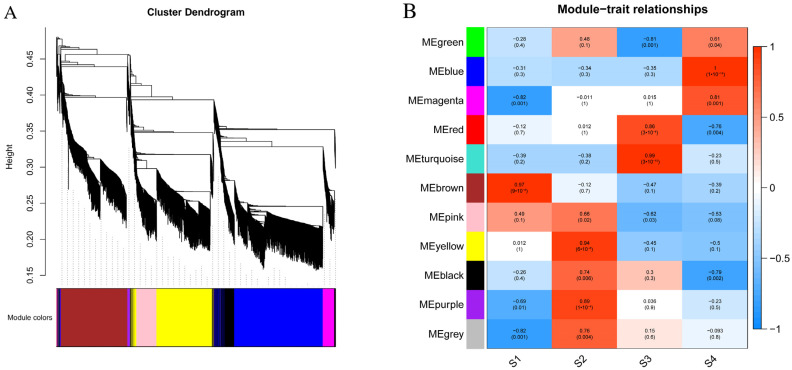
WGCNA analysis. (**A**) Clustering dendrogram of genes, with dissimilarity based on topological overlap, together with assigned merged module colors and theory final module colors. (**B**) Module–trait associations. The color bar indicates the correlation of each module with each sample. Each cell contains the corresponding correlation and *p*-value.

Based on the WGCNA analysis, a total of 6015 genes were categorized into 11 modules, each represented by a distinct color (Figure 5A). With the exception of the grey module, which encompassed nine genes, the gene numbers in each module, delineated based on the gene expression pattern throughout flower development, and ranged from 33 in the purple module to 1304 in the turquoise module. Subsequently, a correlation analysis between the modules and development stages was conducted (Figure 5B). The results indicated that most modules exhibited a positive correlation with developmental stage traits. For instance, seven modules (brown, pink, yellow, black, purple, and yellow) demonstrated significant positive correlations with the S1 and S2 stages, while five modules (green, blue, magenta, red, and turquoise) exhibited significant positive correlations with S3 and S4 stages.

**Figure 6:**
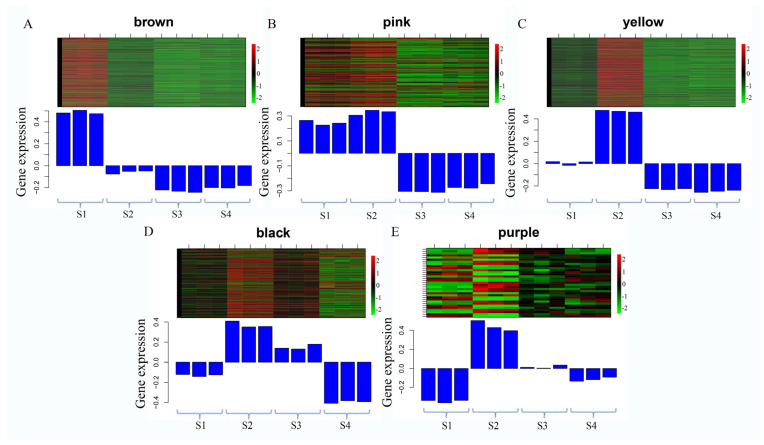
(**A**–**E**) The heatmap of gene expression levels in all samples of selected WGCNA modules. The color bar indicates the relative expression of module genes: red denotes upregulation, and green denotes downregulation.

**Figure 7:**
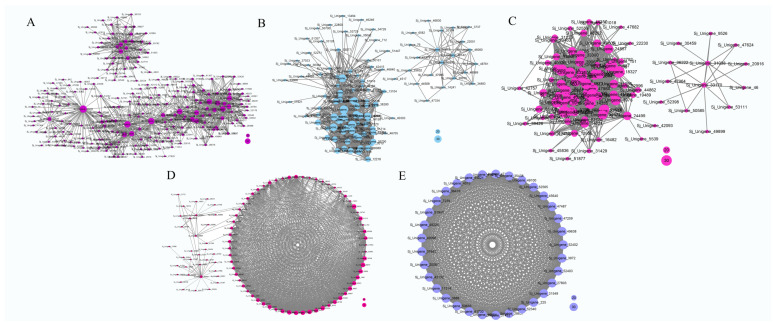
Interaction analyses of the selected modules. The bubble size indicates connection degree of each gene. The networks represented brown, pink, yellow, black, and purple respectively (**A**–**E**).

In the brown module, 862 genes were identified, with the majority showing upregulation in the S1 stage and downregulation in the other three stages (Figure 6A). Functional annotation of these genes revealed the presence of numerous transcription factor (TF) genes, including *bHLH* (Sj_Unigene_42151, Sj_Unigene_44877, Sj_Unigene_48500, Sj_Unigene_40078, Sj_Unigene_52366), *AP2* (Sj_Unigene_49499, Sj_Unigene_43800, Sj_Unigene_51825, Sj_Unigene_32968, Sj_Unigene_52604), *NAC* (Sj_Unigene_47071, Sj_Unigene_48992, Sj_Unigene_23508, Sj_Unigene_18481, Sj_Unigene_34972), *WRKY* (Sj_Unigene_46331, Sj_Unigene_43512, Sj_Unigene_50785, Sj_Unigene_48986), *TIFY* (Sj_Unigene_51942), *MYC2* (Sj_Unigene_20334), *MADS-box* (Sj_Unigene_52863), *MYB* (Sj_Unigene_32451, Sj_Unigene_49803, Sj_Unigene_19281, Sj_Unigene_43132), and *AP2* (Sj_Unigene_51927, Sj_Unigene_49499, Sj_Unigene_43800, Sj_Unigene_49057, Sj_Unigene_51825, Sj_Unigene_32968, Sj_Unigene_52604), suggesting their potential roles in the transcriptome-regulation process. Furthermore, functional genes associated with the regulation of secondary metabolite syntheses were identified in the brown module. These included the *CHS* gene (Sj_Unigene_51046, Sj_Unigene_40370), *PAL* gene (Sj_Unigene_17024, Sj_Unigene_15202), *C4H* gene (Sj_Unigene_39905), *4CL* gene (Sj_Unigene_43373, Sj_Unigene_39561, Sj_Unigene_39873), chalcone reductase (*CHR*, Sj_Unigene_51052), flavonol synthase gene (*FLS*, Sj_Unigene_52689), isoflavone synthase (*IFS*, Sj_Unigene_39498), β-amyrin synthase (Sj_Unigene_9960), Isoflavone 7-O-methyltransferase gene (Sj_Unigene_50922), and *CCR* gene (Sj_Unigene_51830, Sj_Unigene_51903), indicating their potential involvement in secondary metabolism processes. In addition, the brown module contained several other functional genes, including the E3 ubiquitin-protein ligase genes (Sj_Unigene_46831, Sj_Unigene_6463, Sj_Unigene_11257, Sj_Unigene_52773, Sj_Unigene_52610, Sj_Unigene_50371), Flowering time control protein gene (Sj_Unigene_52533), Histone acetyltransferase *HAT* gene (Sj_Unigene_51271, Sj_Unigene_48835, Sj_Unigene_26313, Sj_Unigene_3111), Cytochrome P450 genes (Sj_Unigene_42153, Sj_Unigene_35993, Sj_Unigene_39034, Sj_Unigene_35213, Sj_Unigene_26786, Sj_Unigene_39498, Sj_Unigene_26839, Sj_Unigene_44441), jasmonate O-methyltransferase gene (Sj_Unigene_47110), Jasmonic acid-amido synthetase gene (*JAR1*, Sj_Unigene_28000), Peroxidase gene (Sj_Unigene_52249), and Superoxide dismutase gene (Sj_Unigene_53247). The genes in the brown module may play vital roles in the regulation network (Figure 7A).

In the pink module, 262 genes were identified, with the majority being upregulated during the S1 and S2 stages and downregulated in the S3 and S4 stages (Figure 6B). A functional annotation analysis revealed the presence of several transcription factor (TF) genes, including *WRKY* (Sj_Unigene_30015), *MYB* (Sj_Unigene_51999; Sj_Unigene_50056; Sj_Unigene_43551; Sj_Unigene_50700), *NAC* (Sj_Unigene_38926), and *bHLH* (Sj_Unigene_51762), which likely play crucial roles in the transcriptome-regulation process during the S1 and S2 stages. Additionally, numerous genes associated with secondary metabolism were identified in this module, such as the *PAL* gene (Sj_Unigene_17690), *4CL* gene (Sj_Unigene_30046), *F3H* gene (Sj_Unigene_39212; Sj_Unigene_46830), *FLS* gene (Sj_Unigene_50206), *CHS* gene (Sj_Unigene_44508), squalene monooxygenase gene (*SM*, Sj_Unigene_34883), and *ANR* gene (Sj_Unigene_51762), indicating their potential involvement in secondary metabolite synthesis processes. Moreover, functional genes like *P450* gene (Sj_Unigene_40303), jasmonate-induced protein gene (Sj_Unigene_40068), auxin response factor gene (Sj_Unigene_712), *NRT1* gene (Sj_Unigene_31260), brassinazole-resistant 1 gene (*BZR1*, Sj_Unigene_51714; Sj_Unigene_42860), histone-lysine N-methyltransferase gene (*ATX*, Sj_Unigene_10388), and histone acetyltransferase gene (*HAT*, Sj_Unigene_30588) were also identified (Figure 7B). These collected functional genes may be vital during the S1 and S2 stages.

In the yellow module, the majority of genes exhibited upregulation during the S2 stage, whereas downregulation was observed in the S3 and S4 stages (Figure 6C). Through functional annotation analysis, we identified genes associated with secondary metabolism, including the *CHS* gene (Sj_Unigene_38202) and the caffeoyl shikimate esterase gene (*CSE*, Sj_Unigene_50850). Additionally, several transcription factor genes were detected, such as the *TCP* gene (Sj_Unigene_29761; Sj_Unigene_35974), *WRKY* gene (Sj_Unigene_10573; Sj_Unigene_12354), *MYB* gene (Sj_Unigene_40516), and *SEP* gene (Sj_Unigene_51060; Sj_Unigene_53009), suggesting their potentially vital role in transcriptional regulation. Furthermore, genes related to plant-growth-regulation processes were identified in this module, including the *ARF* gene (Sj_Unigene_10705; Sj_Unigene_2654; Sj_Unigene_4274; Sj_Unigene_5262; Sj_Unigene_3735; Sj_Unigene_4415), Auxilin-related protein gene (Sj_Unigene_42093), and auxin-responsive protein gene (Sj_Unigene_48947; Sj_Unigene_42591; Sj_Unigene_45958), potentially playing a regulatory role in the growth and development of flower buds. Other functional genes observed in this module include probable methyltransferase (*PMT*, Sj_Unigene_27753), *CYP 450* (Sj_Unigene_41376; Sj_Unigene_37517), pectin methylesterase (*PME*, Sj_Unigene_49819; Sj_Unigene_14725), Ubiquitin-conjugating enzyme E2 (*UBC* 5, Sj_Unigene_40175), E3 ubiquitin-protein ligase (Sj_Unigene_39314; Sj_Unigene_21312; Sj_Unigene_2812; Sj_Unigene_46667), jasmonate-induced protein (Sj_Unigene_38256), and *EIN3* (Sj_Unigene_15626), all potentially playing regulatory roles during the S2 stage in the network (Figure 7C).

In the black and purple modules, a total of 279 and 33 genes were identified, respectively. Most of these genes displayed upregulation during the S2 and S3 stages, while downregulation was observed in the S1 and S4 stages (Figure 6D,E). Functional analysis revealed several transcription factor (TF) genes, including *bHLH* (Sj_Unigene_42026, Sj_Unigene_6362, Sj_Unigene_52289, Sj_Unigene_36419), *TCP* (Sj_Unigene_11062), *NAC* (Sj_Unigene_41681), and *MADS*-box (Sj_Unigene_53003, Sj_Unigene_48740, Sj_Unigene_52534) genes. Additionally, functional genes such as *PMT* (Sj_Unigene_16009), *ABA8ox1* (Sj_Unigene_41153), *CYP450* (Sj_Unigene_39947), and E3 ubiquitin-protein ligase (Sj_Unigene_47383, Sj_Unigene_45372, Sj_Unigene_4779) were identified, suggesting potential regulatory roles within the network (Figure 7D,E).

## 4. Discussion

### 4.1. Generation of Comprehensive and High-Quality Transcriptomic Data of S. japonicum

High-quality transcriptome sequences are crucial for advancing molecular breeding efforts in medicinal plants. *S. japonicum*, commonly known as the Chinese scholar tree and widely distributed in China, is a popular medicinal plant. In recent years, single-molecule real-time (SMRT) technology has emerged as an innovative bioinformatics tool [27]. This technology offers longer reading lengths compared to second-generation sequencing techniques [28], eliminating the need for sequence assembly. This advancement has the potential to significantly enhance scientific research, especially for non-model plants rich in genetic information. It improves gene annotation for sequenced species and serves as a valuable reference for unsequenced species [29,30]. In this study, we performed a full-length transcriptome analysis of *S. japonicum* using both SMRT and Illumina sequencing. This approach not only expedites further studies on *S. japonicum* and its relatives but also contributes to a broader understanding of non-model plant genomics. A total of 103.9 GB of raw data was collected, comprising 629,630 circular consensus sequences (CCS) and 525,408 full-length non-chimeric (FLNC) reads. The obtained unigenes had an average length of 2421 bp, with a high annotation rate of 99.5% for *S. japonicum*. The majority of these unigenes exhibited alignment to closely related bean plants, specifically Glycine max and Lupinus angustifolius. These findings represent the first comprehensive insight into the full-length transcriptome of *S. japonicum*, offering an effective method to identify candidate genes associated with flavonoid biosynthesis.

### 4.2. Function of Genes Involved in Flavonoid Biosynthesis

The flowers and flower buds of *S. japonicum* have been recognized for their rich flavonoid content and medicinal properties in Asia, particularly for their antioxidative properties [31]. These attributes, coupled with the diverse secondary metabolites present, have positioned *S. japonicum* flowers as economically significant crops in various regions of China [32,33]. Previous studies have highlighted the predominant presence of flavonoids in *S. japonicum* compounds [34,35], which encompass a broad family of secondary metabolites in plants, including flavonols, flavones, isoflavones, dihydroflavonols, leucoanthocyanidins, and chalcone, serving as the precursor of flavonoids [36,37]. A wealth of research has elucidated the physiological and pharmacological activities associated with flavonoid compounds, showcasing their role in mitigating reactive oxygen species (ROS) in vivo and inhibiting the development of various diseases [38]. Pharmacological studies have suggested the potential of *S. japonicum* flowers to treat a variety of diseases and aid in organismal repair [39,40].

Flavonoids are synthesized through the phenylpropanoid pathway, originating from phenylalanine, a product of the shikimate pathway [41]. The initial key enzyme in the phenylpropanoid pathway is PAL, which catalyzes the deamination of phenylalanine to trans-cinnamic acid. PAL plays a crucial role in regulating carbon flux from the primary to secondary metabolism in plants [42,43]. In this study, most detected *PAL* genes were upregulated in the S1 and S2 stages, indicating their potential importance in the synthesis of secondary metabolites and the increase in flavonoid content during the flower bud stage.

The C4H enzyme is pivotal in the second key step of the phenylpropanoid metabolism pathway, converting trans-cinnamic acid to coumaric acid [44]. Meanwhile, 4CL, a critical enzyme in the phenylpropanoid pathway, acts as the main branch point, converting p-coumaric acid, ferulic acid, and caffeic acid into their corresponding CoA esters [45]. Both *C4H* and *4CL* genes play a role in flavonoid metabolism [44,45,46]. For instance, 4CL is essential for flavonoid synthesis [47], and its activity can impact the content of anthocyanins and flavonols in plants [48]. In this study, a *C4H* gene and several *4CL* genes were identified. Gene expression analysis revealed an upregulation of the *C4H* gene in the S3 stage and downregulation in the S1 stage. Conversely, most *4CL* genes were upregulated in the S1 stage and downregulated in the other three stages. This suggests that, in the process of flavonoid synthesis in *S. japonicum* flower buds, *4CL* may play a more significant role than *C4H*.

Chalcone, a primary intermediate in the flavonoid metabolism pathway, serves as an essential structural foundation for subsequent flavonoid synthesis, emphasizing the significance of genes involved in chalcone synthesis [49,50]. Previous research has highlighted the *CHS* gene, encoding the corresponding enzyme that catalyzes chalcone formation [49]. The CHI enzyme further converts chalcone into the corresponding flavanone, a precursor for the synthesis of various flavonoid compounds, including flavonoids, isoflavones, flavonols, and anthocyanins [51]. Additionally, enzymes such as F3H, F3′H, and FLS are involved in flavonol and flavanone synthesis [52]. LDOX, LAR, and ANR, affecting anthocyanin biosynthesis and proanthocyanidin content levels in plants, also contribute to the intricate process of flavonoid metabolism [53,54,55]. In the S1 and S2 stages, most *CHS*, *LDOX*, and *LAR* genes were upregulated, while some *CHI* and *FLS* genes showed no significant upregulation. These results suggest that the genes related to chalcone, anthocyanins, and proanthocyanidins biosynthesis play a key role in the S1 and S2 stages of *S. japonicum* flowers. Notably, during the S1 and S2 stages, all detected genes related to *F3H*, *F3′H*, and *ANR* displayed upregulation, indicating their pivotal role in facilitating the biosynthesis of proanthocyanidin content and dihydroflavonols, including dihydrokaempferol, dihydroquercetin, and dihydromyricetin, during the flower bud stage, while further exploration of the quantitative relationship between the activity of enzymes like PAL, C4H, and 4CL and the flavonoid content in *S. japonicum* flowers could provide insights into how these enzymes precisely regulate flavonoid synthesis.

### 4.3. The Candidate Transcription Factors Might Regulate Flavonoids Biosynthesis in S. japonicum Flower

Transcriptional regulation plays a pivotal role in modulating flavonoid biosynthesis in plants, with transcriptional regulator factors such as MYB, bHLH, and WRKY known to be involved in flavonoid biosynthesis [56,57].

MYB transcription factors (TFs) belong to plant-specific TFs and are key regulators of structural genes in flavonoid-related biosynthesis pathways, a topic widely discussed in the literature [58,59]. Numerous MYB TFs have been identified for their specific role in increasing expression levels or activating related genes in the flavonoid synthesis pathway, such as *CHS*, *F3H*, *DFR*, *LAR*, and *ANS* [60,61,62]. For instance, the overexpression of PpMYB17 in pear calli and *Arabidopsis* upregulated the expression of flavonoid biosynthesis-related genes, particularly *FLS*. Metabolite quantification analysis revealed significant differential accumulation of flavonoid-related metabolites, including flavonols, flavanones, flavones, and isoflavones, in the overexpressing calli compared to wild-type calli [63]. In *Erigeron breviscapus*, MYB TF was identified as a positive regulator of flavonoid biosynthesis by activating the expression levels of *FLS*, *CHI*, *CHS*, and *F3H* genes, directly related to flavonoid biosynthesis [64]. A previous study characterizing the *MYB* gene family in *Zanthoxylum bungeanum* provided new insights, suggesting that MYB TFs may regulate flavonoid metabolism with temporal–spatial changes in individual growth and maturation [3]. In our study, numerous *MYB* TF genes were identified and expressed across different flower development stages. Co-expression analysis revealed four *MYB* TF genes in both the brown and pink modules, and six in the yellow module. Within these modules, several structural genes related to flavonoid synthesis were observed, including the presence of the *CHS* gene. Moreover, both the brown and pink modules housed the *PAL*, *4CL*, and *FLS* genes. Additionally, the brown module exclusively contained the *CHR* gene, while the yellow module exclusively showcased the *F3H* and *F3’H* genes. These *MYB* TF genes may potentially regulate these functional genes, playing a crucial role in the regulatory network governing flavonoid synthesis.

bHLH TFs have been extensively studied for their role in regulating flavonoid synthesis in plant species and various other plant-growth and -development pathways [65,66]. Previous studies have shown that some bHLH TFs serve as essential activation partners for MYB TFs, enhancing the activity of conserved cis-regulatory element promoters in flavonoid and anthocyanin biosynthesis genes [67]. For example, in Chrysanthemums, bHLH TF significantly activated the transcription of the *DFR* gene, triggering anthocyanin accumulation [68]. The *VvbHLH* gene in grapes was identified as potentially related to flavonoid synthesis [69]. Heterogeneous overexpression of the *PabHLH* gene elevated both flavonoid and anthocyanin synthesis in Arabidopsis by upregulating structural genes involved in flavonoid synthesis [70]. In our study, we identified two *bHLH* genes in the black module, five in the brown module, one in the purple module, and three in the yellow module. In addition to numerous functional genes directly associated with the flavonoid synthesis process, other genes with regulatory functions, including the *PMT* and E3 ubiquitin protein ligase genes, were also identified within these modules. These functional genes, crucial for flavonoid synthesis, may be subject to coordinated regulation by both bHLH transcription factors and other regulatory genes.

Co-expression-network prediction and cis-element analysis with the promoters of flavonoid biosynthesis-related structural genes suggested that WRKY and AP2 TFs may play vital roles in regulating flavonoid biosynthesis [71]. Transcriptomic and spatiotemporal expression characteristic analysis in Freesia indicated that the expression patterns of selected *WRKY* and *AP2* TF genes were consistent with flavonoid accumulation in the petals, suggesting their involvement in the regulation of flavonoid metabolism [72]. In our study, a total of five *WRKY* genes were identified in the brown module, one in the pink module, and three in the yellow module. Additionally, the brown module was found to contain eight *AP2* genes. These identified TF genes likely code for proteins involved in regulating the flavonoid synthesis process, collaborating with other TFs within their respective modules to establish a comprehensive and interconnected regulatory network. In-depth investigations into the regulatory mechanisms through which transcription factors such as MYB, bHLH, and WRKY influence the expression of downstream genes in flavonoid biosynthesis, including the elucidation of interactions and regulatory networks among these transcription factors, would provide valuable insights. We should explore how genetic modification or cultivation techniques can be used to increase the flavonoid content in *S. japonicum* flowers to enhance their medicinal value and economic potential.

## 5. Conclusions

In this study, we have successfully generated a comprehensive and valuable full-length transcriptome database of *S. japonicum*, marking the first of its kind. A total of 53,281 high-quality full-length transcripts were produced, with the number of differentially expressed genes (DEGs) gradually increasing across flower development stages. Additionally, through functional gene analysis and weighted gene co-expression network analysis (WGCNA), we have identified candidate genes associated with flavonoid synthesis, along with regulatory genes. Moreover, our study has brought to light newly discovered transcription factors (TFs) that may play a crucial role in the regulation of flavonoid synthesis in the flower buds of *S. japonicum*. In conclusion, our research not only provides a valuable genetic resource for investigating flavonoid biosynthesis in *S. japonicum* but also holds the potential to reveal novel candidate transcription factors involved in regulating flavonoid synthesis in *S. japonicum* petals. The implications of this research extend beyond the immediate findings, establishing a robust foundation for future investigations into the molecular mechanisms and gene functions underlying flower development in *S. japonicum*.

## Figures and Tables

**Table 1:** The number and percentage of annotated genes in different databases.

Database	Annotated Number	Percentage
NR	25,261	0.99
SwissProt	22,482	0.88
KEGG	25,175	0.99
KOG	16,474	0.65
GO	18,952	0.75
NT	25,116	0.99
Pfam	18,952	0.75

**Table 2:** Statistics of differentially analysis genes in different groups.

Comparison Groups	Total Number	Upregulated	Downregulated
S1-vs.-S2	7964	5048	2916
S1-vs.-S3	11,824	6570	5254
S1-vs.-S4	12,768	7576	5192
S2-vs.-S3	12,804	6558	6246
S2-vs.-S4	12,278	6895	5383
S3-vs.-S4	13,215	7439	5776

**Table 3:** Selective clusters with diverse patterns in flower development of *S. japonicum*.

Cluster Name	Enrichment Pathway Name	Gene Number	Gene Expression Patterns
Cluster 2	Base excision repair	41	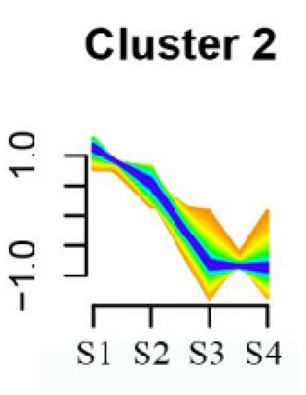
	Butanoate metabolism		
	Valine, leucine, and isoleucine biosynthesis		
	Linoleic acid metabolism		
	Homologous recombination		
	Synthesis and degradation of ketone bodies		
	Monobactam biosynthesis		
	Glycosphingolipid biosynthesis—ganglio series		
	Flavonoid biosynthesis		
Cluster 5	α-Linolenic acid metabolism	172	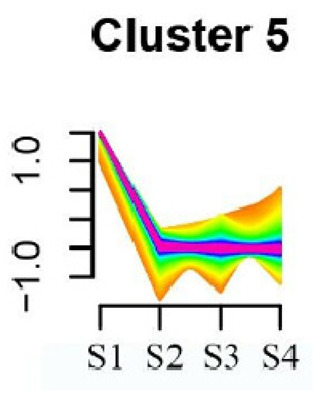
	Glycolysis/Gluconeogenesis		
	Tyrosine metabolism		
	Fatty acid degradation		
	Taurine and hypotaurine metabolism		
	Isoflavonoid biosynthesis		
	Galactose metabolism		
	Linoleic acid metabolism		
	Cutin, suberine and wax biosynthesis		
	Ubiquinone and other terpenoid-quinone biosynthesis		
	Peroxisome		
	Pentose phosphate pathway		
Cluster 7	Aminoacyl-tRNA biosynthesis	24	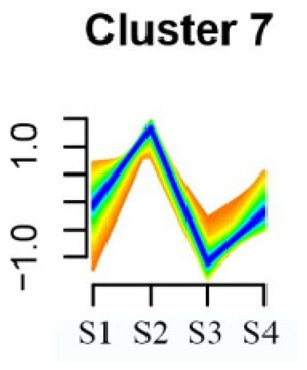
	Porphyrin and chlorophyll metabolism		
	Diterpenoid biosynthesis		
	Vitamin B6 metabolism		
	Ubiquinone and other terpenoid–quinone biosynthesis		
Cluster 9	Spliceosome	58	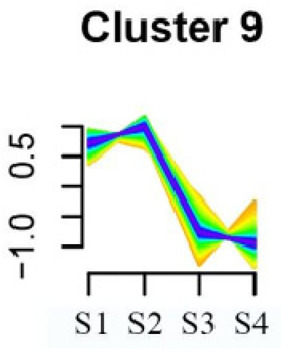
	Linoleic acid metabolism		
	Isoquinoline alkaloid biosynthesis		
	Flavonoid biosynthesis		
	Other glycan degradation		
	Sphingolipid metabolism		
	Carbon fixation in photosynthetic organisms		
Cluster 13	Fatty acid biosynthesis	69	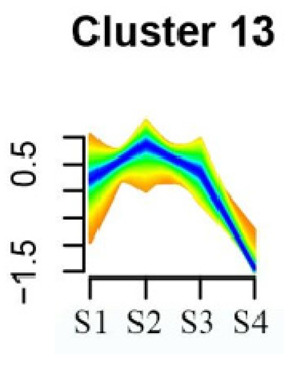
	Biotin metabolism		
	Starch and sucrose metabolism		
	Amino sugar and nucleotide sugar metabolism		
	Steroid biosynthesis		
	Tryptophan metabolism		
Cluster 18	Linoleic acid metabolism	62	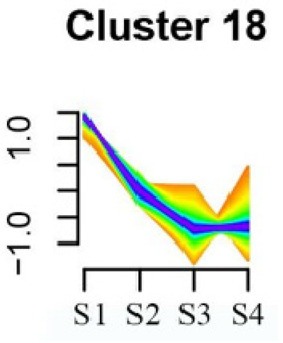
	DNA replication		
	α-Linolenic acid metabolism		
	Spliceosome		
	Flavonoid biosynthesis		
	Phenylpropanoid biosynthesis		
	Pyrimidine metabolism		

## Data Availability

The clean data in this study are accessible to the Nucbank database (https://ngdc.cncb.ac.cn/search/specific?db=bioproject&q=PRJCA021261, accessed on 13 November 2023) with the BioProject accession number PRJCA021261.

## Supplementary Materials

The following supporting information can be downloaded at: https://www.mdpi.com/article/10.3390/genes15030329/s1, Figure S1: Functional classification of COG database; Figure S2: GO functional classification; Figure S3: KEGG functional classification; Figure S4: The overlap of expressed genes in each flower development stage; Table S1: The result of clean data of full-length transcriptome; Table S2: The result of CCS sequence; Table S3: The result of FLNC; Table S4: Total of 20 distinct clusters of different gene expression patterns.
