# Supplementary material for: Full-Length Transcriptome Sequencing and Comparative Transcriptomic Analyses Provide Comprehensive Insight into Molecular Mechanisms of Flavonoid Metabolites Biosynthesis in Styphnolobium japonicum
Source: Genes (Basel). 2024 Mar 3;15(3):329. doi: 10.3390/genes15030329 (PMC10970609; doi:10.3390/genes15030329)
Supplement: Supplementary file 1 [file genes-15-00329-s001.zip › Figure S3.pdf]

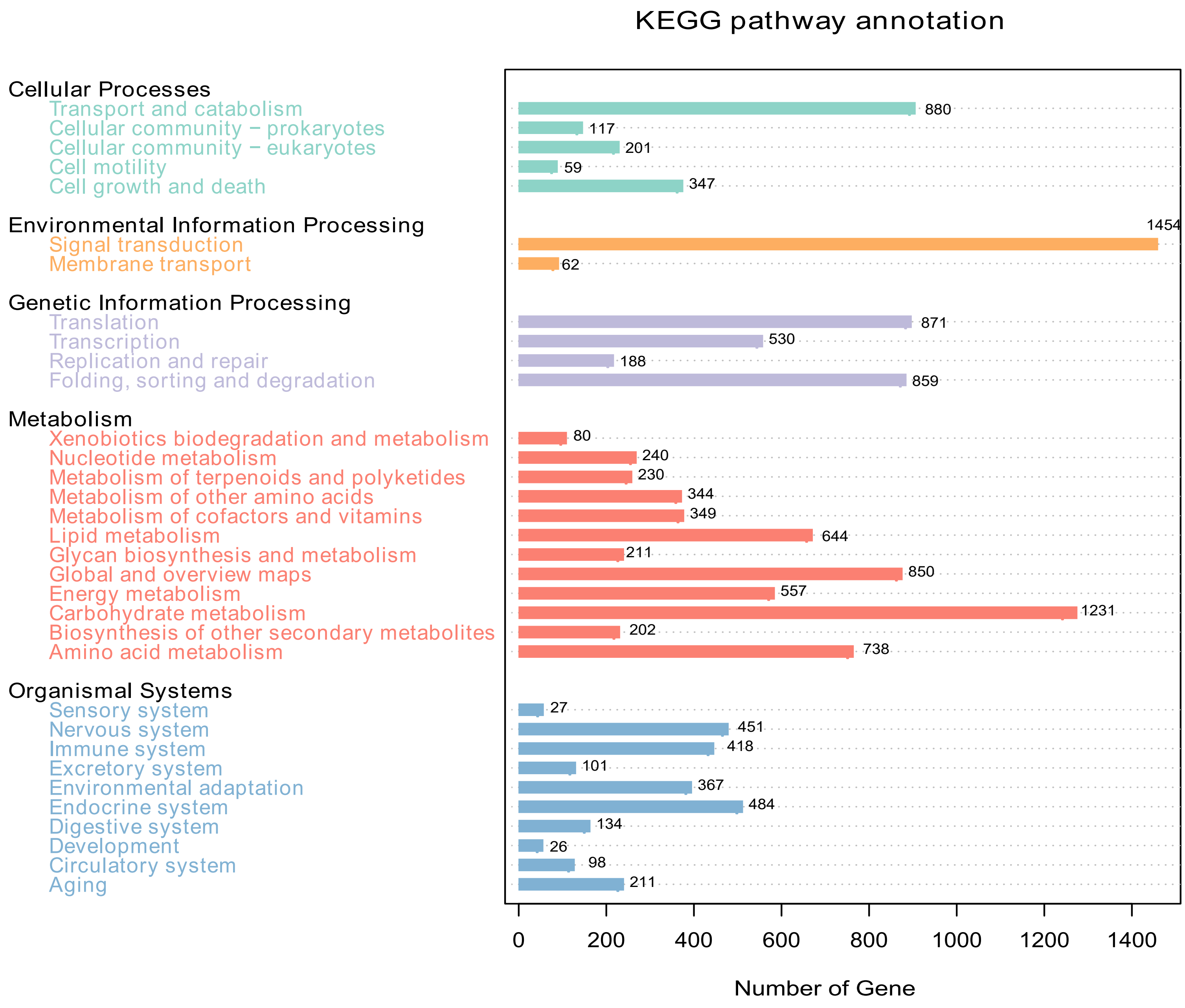
KEGG pathway annotation
Cellular Processes
Transport and catabolism
Cellular community − prokaryotes
Cellular community − eukaryotes
Cell motility
Cell growth and death
Environmental Information Processing
Signal transduction
Membrane transport
Genetic Information Processing
Translation
Transcription
Replication and repair
Folding, sorting and degradation
Metabolism
Xenobiotics biodegradation and metabolism
Nucleotide metabolism
Metabolism of terpenoids and polyketides
Metabolism of other amino acids
Metabolism of cofactors and vitamins
Lipid metabolism
Glycan biosynthesis and metabolism
Global and overview maps
Energy metabolism
Carbohydrate metabolism
Biosynthesis of other secondary metabolites
Amino acid metabolism
Organismal Systems
Sensory system
Nervous system
Immune system
Excretory system
Environmental adaptation
Endocrine system
Digestive system
Development
Circulatory system
Aging
880
117
201
59
347
1454
62
871
530
188
859
80
240
230
344
349
644
211
850
557
1231
202
738
27
451
418
101
367
484
134
26
98
211
0
200
400
600
800
1000
1200
1400
Number of Gene
