# Supplementary figures and images for: Full-Length Transcriptome Sequencing and Comparative Transcriptomic Analyses Provide Comprehensive Insight into Molecular Mechanisms of Flavonoid Metabolites Biosynthesis in Styphnolobium japonicum
Source: Genes (Basel). 2024 Mar 3;15(3):329. doi: 10.3390/genes15030329 (PMC10970609; doi:10.3390/genes15030329)

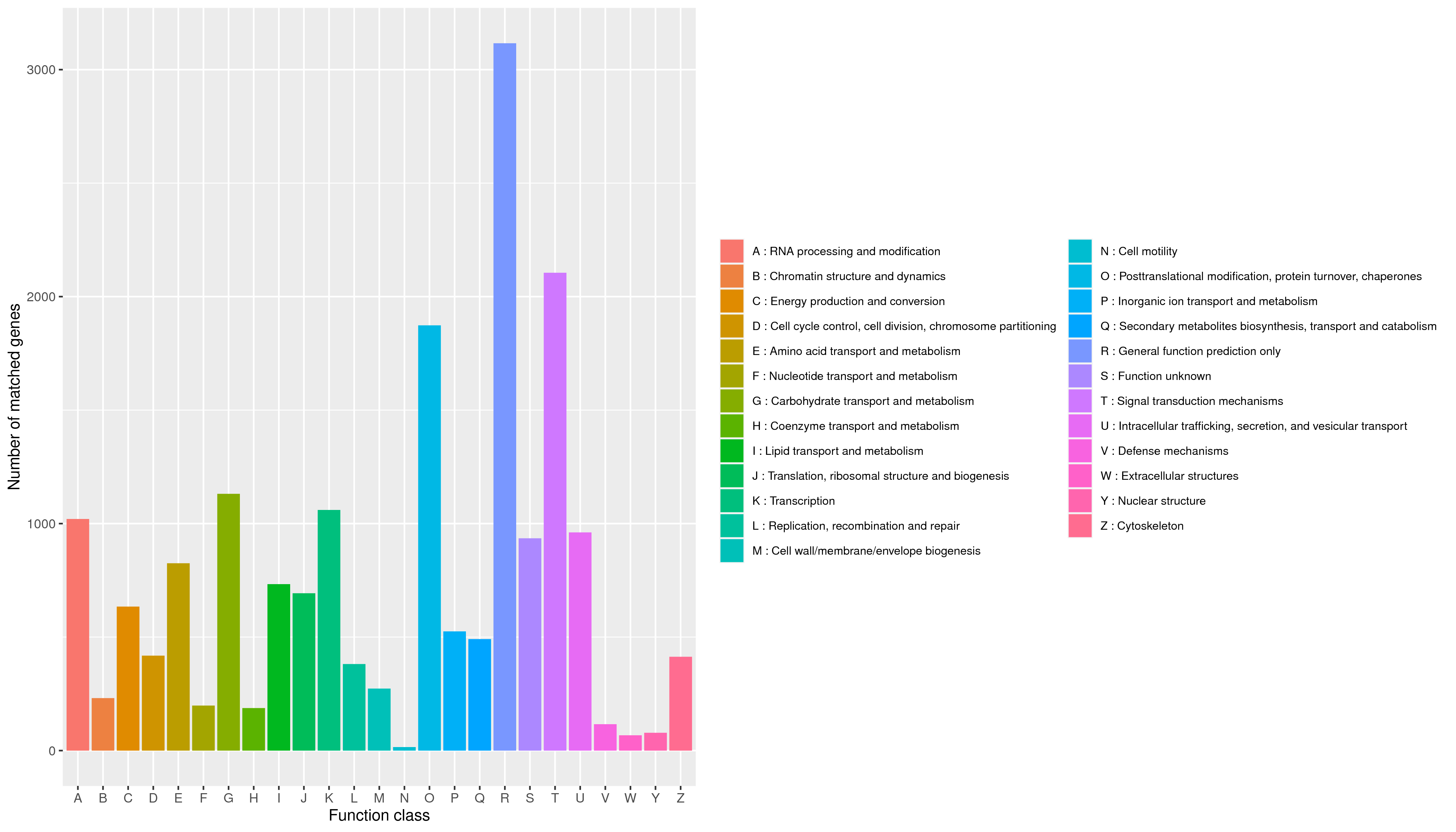

Supplement: Supplementary file 1 [file genes-15-00329-s001.zip › Figure S1.png]

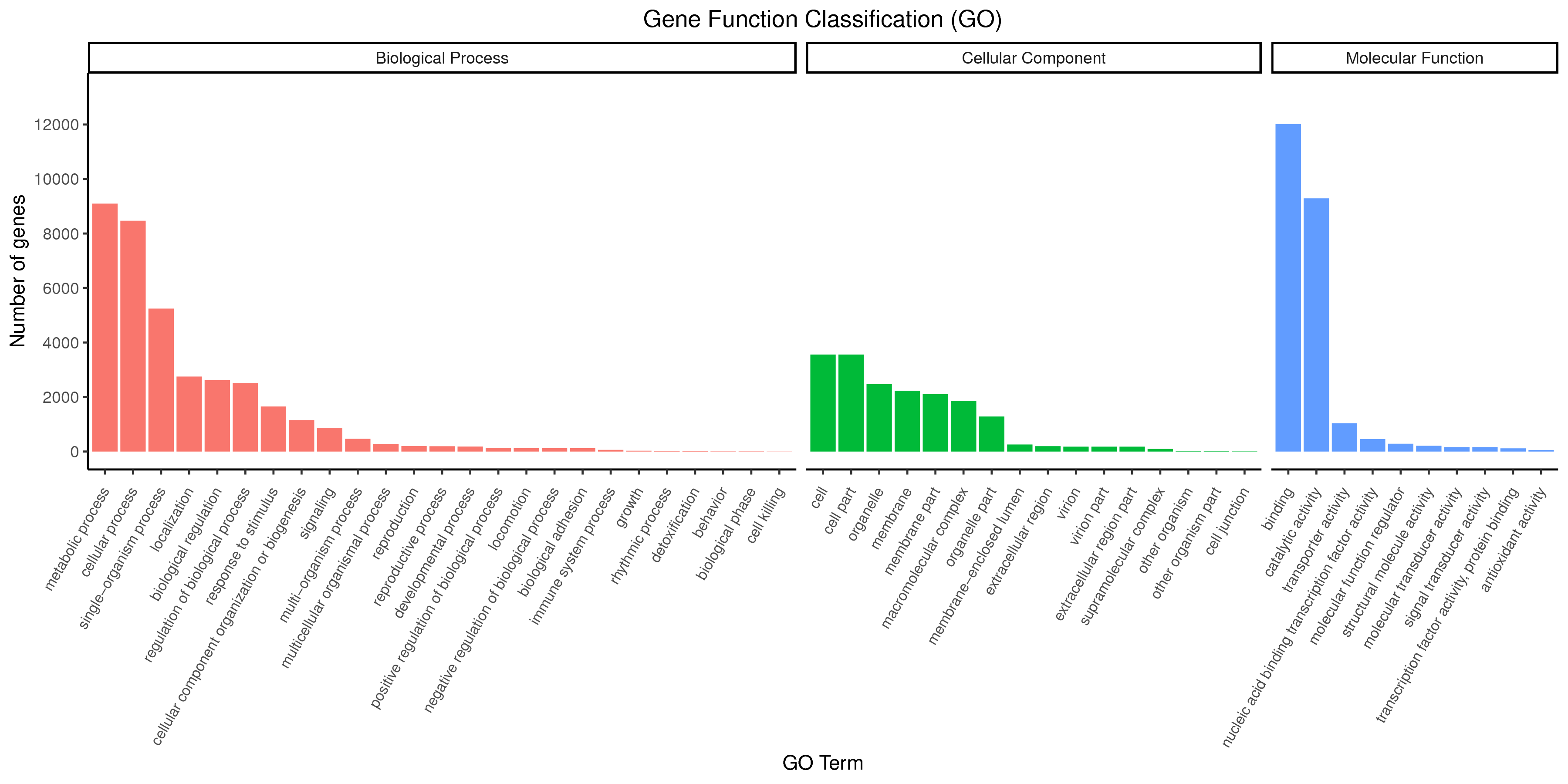

Supplement: Supplementary file 1 [file genes-15-00329-s001.zip › Figure S2.png]

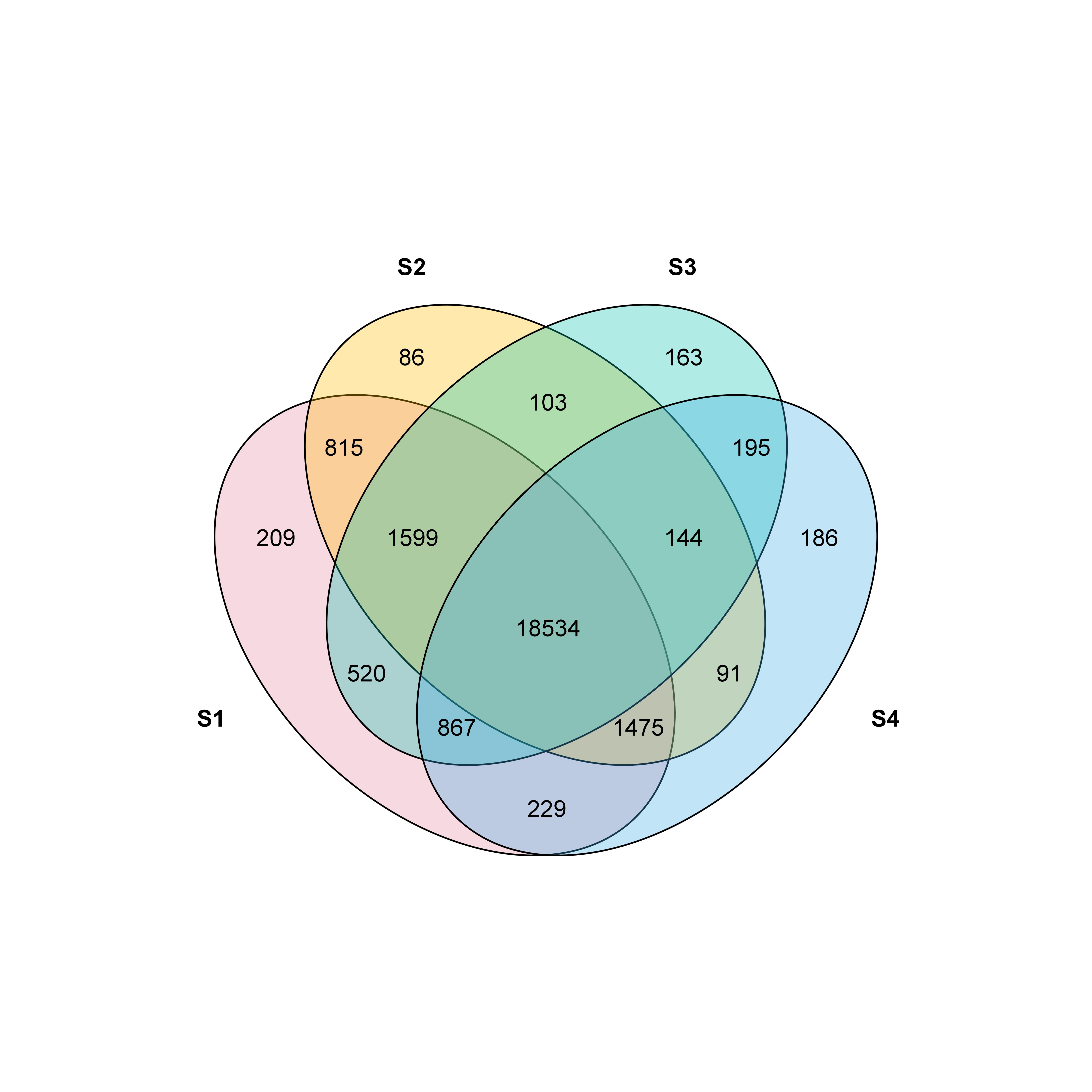

Supplement: Supplementary file 1 [file genes-15-00329-s001.zip › Figure S4.jpg]
